# The Application of Digital Platforms in Supporting UK International Medical Graduates

**DOI:** 10.7759/cureus.10750

**Published:** 2020-10-01

**Authors:** Mina Farag, Idowu Olaogun

**Affiliations:** 1 General Practice, Health Education England (West Midlands), Birmingham, GBR; 2 Medicine, California Institute of Behavioral Neurosciences & Psychology, Fairfield, USA; 3 Endocrinology, University of Ibadan College Hospital, Ibadan, NGA

**Keywords:** uk imgs, overseas doctors, nhs, induction, doctors in training

## Abstract

Although international medical graduates (IMGs) constitute considerable percentage of doctors in the National Health Service (NHS), they face several challenges in acclimatizing to the NHS health system. Communication skills, language subtleties, and career progression difficulties are among the most important challenges that overseas doctors face.

Some resources are already available to support these doctors and several trusts across the UK have developed local interventions and educational programs to help their doctors bridge the gaps in their knowledge. However, there is no proof of the external validity of these programs and none are identified as effective on a national level.

Senior IMGs are leading very popular and inspiring projects using digital platforms, especially social media. We identified several social media pages, groups, and websites subscribed to by hundreds of thousands of doctors in the UK and around the world, including doctors who are planning a future career in the UK. These platforms provide information, resources, support, and answers to questions posed by junior IMGs.

Inspired by these projects and also by an Australian project that transformed a local IMG education program, we studied whether using digital platforms and transforming evidence-based local programs to national ones would be the best way forward to support IMGs.

## Introduction and background

International medical graduates (IMGs) are a very important group of doctors in the National Health Service (NHS) workforce. About 27% of doctors did not gain their medical degree from the UK or European medical school [1]. Most overseas junior doctors gain their General Medical Council (GMC) license to practice after successfully passing an English exam and Professional and Linguistic Assessments Board (PLAB) tests. According to the GMC website, from 2013 to 2018, over 11,000 doctors passed the second and last PLAB exams, allowing them to apply for GMC registration [2].

These doctors add invaluable support to the NHS system. But unfortunately, starting a new job in a different health care system is not an easy task. It is well documented in the literature that several challenges facing IMGs in the UK make their transition to the host health care system difficult. Challenges such as language, communication skills, work culture, passing exams, local guidelines, and health care traditions make the acclimatization process very stressful [3, 4].

Around the UK, many interventions and initiatives have been put in place to try to tackle this problem and to support new doctors, especially in their first job in the UK [5]. Most of these interventions were designed locally with limited evidence on their external validity [6]. Despite having a limited effect, they do reflect that a lot of trusts around the UK feel the need for such programs.

Being well aware of the problem, overseas doctors usually form associations and join social media groups to help each other. Social media appears to be the favorite method of communication between IMGs to discuss their learning needs and to form online resources to help each other fill the gaps in their knowledge. For example, there are several PLAB study groups on social media platforms, and one of these is so popular that it now has over 200,000 subscriptions from doctors around the world [7].

This article discusses the problems faced by IMGs, investigates some current interventions, and sheds light on the significant role that digital platforms, especially social media, play in helping IMGs. The surprising success of social media from this perspective should be inspiring for all medical educators in the future to consider producing online resources that can be used on social media and could be accessed around the world by doctors considering a move to the UK in the future.

## Review

Methods

The literature review used PubMed®, PubMed Central®, and Google Scholar to find relevant research. The keywords used were "IMGs UK", "NHS", "induction" and "overseas doctors". The research included articles describing teaching programs for IMGs and the challenges facing them. Exclusion criteria included articles that discussed political aspects (Brexit, points of view about health professional migrations, etc.) and articles that were not written in English. Initial research included hundreds of articles, but this particular review depended mainly on 31 articles that were found to be the most current, relevant to UK practice, and avoided politics.

Language and communication skills

Being aware of language skills' impact on IMGs' relationship with patients and colleagues, the GMC always includes evidence of language knowledge in the process of registering new doctors within the UK [8]. When preparing this review, it was discovered that language and communication skills are among the most challenging issues facing IMGs in their new life in the UK [9, 10].

Cultural differences can also impact the doctor-patient relationship. For example, doctors who come from religious communities might have broken bad news or communicated difficult decisions using a religious mindset with frequent references to God [9]. However, this practice is not generally used by doctors in Britain as large sectors of the community identify as atheist or non-religious [11].

When compared to other countries around the world, the doctor-patient relationship is different in the UK. Involving the patient in making decisions regarding their health is one of the most important objectives that clinicians have to achieve to deliver patient-centered care. However, in different societies, this is not the case as it might be acceptable for doctors to make decisions without giving the patient enough information and with less emphasis on building rapport with the patient [3]. These concepts are practiced by and are well known to senior IMGs, but it might take some time for new doctors to embrace the same attitude and concepts.

Even when new overseas doctors try to deliver patient-centered care, they are sometimes hindered by language subtleties [12]. Almost all the studies considered in this review mentioned that language subtleties are one of the most important factors affecting doctors' communication skills, and already, some training programs have been developed to address the need to improve their use of the English language [12, 13].

In 2017, a German systematic review about intercultural challenges faced by IMGs summarised the above-mentioned points by finding that they mostly struggled with shared decision making and patient-centered care, subtleties of the foreign language, and the organizational structures of the host medical system [14].

Education and career progression challenges

Overseas doctors struggle more than UK graduates with passing exams, which impacts their career progression [3,15-17]. In this respect, the literature focused on exams from the Membership of the Royal College of General Practitioners (MRCGP), the applied knowledge test (AKT), and clinical skills assessment (CSA). In a semi-structured qualitative study exploring why IMGs perform less well in AKT exams, it was found that they struggle more with theoretical questions that are not directly related to day-to-day practice [16]. Additionally, different undergraduate training, being less familiar with UK guidelines, and language barriers were among factors affecting their performance.

An interesting study was conducted using data from more than 1,000 IMG general practitioners (GP) training applicants from 2008 to 2012 [3]. To apply for GP training, candidates have to pass two exams: the situational judgment test (SJT) and the clinical problem-solving test (CPST). The study found that CSA exam scores were more strongly predicted by SJT scores than by performance on the CPST. To bridge this gap, IMGs need tailored education programs addressing interpersonal communication skills and topics related to British culture, NHS guidelines, and structure. Based on this study, addressing these issues is more important in helping IMGs to pass exams, rather than improving general medical knowledge.

Moreover, IMGs are also more likely to struggle with specialty training and getting satisfactory outcomes in their annual review of competence progression (ARCP) [18, 19]. In an observational study involving more than 50,000 UK trainee doctors, more than 11,000 of them were IMGs. It was revealed that most doctors who registered with the GMC via the PLAB pathway were more likely to obtain suboptimal outcomes in ARCP when compared to their UK peers [18].

Another study involved struggling IMG doctors in training, which was done on a smaller scale and involved overseas pediatrics trainees in Rotherham, Sheffield, and Doncaster [19]. This study identified multiple barriers faced by those doctors, including a lack of information about the NHS, difficulties in team working, preparing for Royal College exams, local and colloquial words, different accents, and communicating sensitive issues.

'That's your patient. There's your ventilator.'

This eye-catching headline was the title of a study on difficulties faced by the European Economic Area (EEA) anesthetists working in a London hospital in the process of acclimatization to UK practice norms [20]. This review focused on the support needed for IMGs. However, it is worth mentioning that even EEA graduates faced similar problems.

Available support for IMGs

Currently, several resources are available for help [21]. Traditionally, available support is conveyed through formal inductions, supervisors, and official websites. The GMC provides UK practice workshops for overseas doctors [22]. Additionally, most trusts have induction programs for new starters, and a lot of trusts provide work shadowing for doctors starting their first job in the UK [23]. Furthermore, some IMGs find clinical attachments before the start of their first job to be helpful [24]. The support of educational supervisors is also crucial, especially in the first transition period [25].

There are also online resources. E-learning for healthcare provides an e-learning program to cover social, legal, and ethical aspects of working in the UK [26]. Some doctors find the GMC's good medical practice webpage useful [27].

Several interventions were identified in the literature, including teaching programs to improve language and communication skills. However, they were local programs that helped a small number of doctors in a few hospitals. Among those were a communication training program in Dumfries and Galloway Royal Infirmary and another in the North West, where the expertise of a combination of language and clinical tutors was used [28, 29].

REFRESH project

Two researchers from the University Hospital of Coventry and Warwickshire created a project called REFRESH (revise and enhance from roleplay ethical scenarios in hospital) [30]. With the help of six overseas doctors, they used high-fidelity simulations to design a course for IMGs managing common medical, ethical, and legal challenges within the NHS; this course was then included in the hospital induction program.

Educational programs

North West Health Education England developed an educational program for GP trainees who had previously failed their CSA exam [13]. Results after the course were encouraging for IMGs who joined the course. The success of the program was put down to addressing the learning needs of participants as they used learning tools written by experts to reappraise their learning needs. They also had help from experienced educators, who worked with trainers and trainees to design education plans, which was of paramount importance. 

Is that enough? Inspiration for the future

As previously mentioned, there are some resources to help IMGs, but not enough to tackle the issues as IMGs are still struggling [4, 9, 31, 32]. Current interventions in the UK are on a small scale, serving a small number of doctors. Furthermore, some available resources are generic and not tailored to overseas doctors' needs [6].

In the next section, we will highlight two inspiring interventions, one by senior IMGs and one Australian study suggesting that digital platforms could transform a local evidence-based program into a successful national project.

Could the answer be social media? Inspiring efforts by senior IMGs

Facing all the previously mentioned challenges, international medical graduates form social media groups, websites, and associations meet, share experiences, and help each other. These groups are full of resources written by senior IMGs to help juniors to get accustomed to the British health care system and to prepare them for exams even before coming to the UK. 

There are several groups. Table 1 mentions only a few of them as an example and shows the popularity of these social media platforms. 

**Table 1:** Some of the current digital platforms to support IMGs PLAB - Professional and Linguistic Assessments Board; IMGs - international medical graduates; GMC - General Medical Council *Numbers of subscribers in December 2019

Name	Social media type	Number of subscribers*	Details
PLAB [33]	Facebook group	More than 200,000	This group provides the resources needed for the PLAB exam. Subscriptions are from doctors around the world who are planning to move to the UK.
International medical graduates in the UK [34]	Facebook	More than 88,000	A group for general discussions and resources for support. Usually, the advice is given from senior IMGs to junior IMGs.
Road to the UK [35]	Website, Instagram, Facebook page, YouTube channel	More than 1,000; more than 30,000; more than 5,000	Website and social media groups about the pathways to register with the GMC as a doctor and the different experiences of working in the UK.
Egyptian Doctors in UK [36]	Facebook group	More than 19,000	A group for Egyptian doctors providing advice on working in the UK and support from senior Egyptian IMGs.

IMGs prefer to communicate on social media, and it is an important source for doctors who are not yet in the UK but seek a career in the UK in the future; this is evident by the huge number of subscriptions to social media groups. The future of IMG training programs is about using these platforms in a more organized and official way or by developing a more formal, evidence-based digital platform for different IMG education programs.

'Doctors speak up' - inspiring project from Australia

In 2014, a group of researchers in Australia conducted a project that aimed to help IMGs improve their communication skills. Through video-recorded consultations with volunteer IMGs, they found that many participants tried to deliver patient-centered care but, according to the study, were hindered by what they described as 'limited interactional competence to elicit information and negotiate behaviors as well as a limited repertoire of English grammar, vocabulary and phonological phrasing for effective interaction' [12]. Based on this study, they developed 'Doctors speak up' - an online multimedia resource to address these issues [37]. The website provides idioms, transcripts of conversations, and other resources. The website was so successful that it had 19,500 visitors between March 2012 and November 2013.

Recommendations

Given the scale of the problem, there is a need for a national-scale effort to organize evidence-based teaching programs tailored to IMGs' needs. Using digital platforms, especially social media, in an organized way could be the best way forward because they are very popular among the target group. Social media is also easily accessible at any time from anywhere in the world, so that oversees doctors could use them even before arriving in the UK to prepare for a new life in a different country.

## Conclusions

UK IMGs struggle to become accustomed to UK health care practices. Literature shows that communication and language issues are among the most prominent challenges facing overseas doctors. They also struggle more with exams and consequently, career progression. To tackle this problem, many local small-scale projects were identified and revealed the need for larger, national projects to provide the needed support for IMGs. Recommended solutions have also been highlighted in this article.
